# STAR mapping method to identify driving sites in persistent atrial fibrillation: Application through sequential mapping

**DOI:** 10.1111/jce.14201

**Published:** 2019-10-03

**Authors:** Shohreh Honarbakhsh, Richard J. Schilling, Malcolm Finlay, Emily Keating, Waqas Ullah, Ross J. Hunter

**Affiliations:** ^1^ Electrophysiology department The Barts Heart Centre London United Kingdom

**Keywords:** atrial fibrillation, atrial tachycardia, catheter ablation, drivers, mapping

## Abstract

**Introduction:**

The optimal way to map localized drivers in persistent atrial fibrillation (AF) remains unclear. The objective of the study was to apply a novel vector mapping approach called Stochastic Trajectory Analysis of Ranked signals (STAR) in AF.

**Methods and Results:**

Patients having persistent AF ablation were included. Early sites of activation (ESA) identified on global STAR maps created with basket catheters were used to guide AF ablation post‐pulmonary vein isolation (PVI). All patients also had sequential STAR maps created with ≥10 PentaRay recordings of 30 seconds. These were validated offline in their ability to identify the ESA targeted with a study‐defined ablation response (AF termination or cycle length [CL] slowing of ≥30 ms).

Thirty‐two patients were included in whom 92 ESA were identified on the global STAR maps, with 73 of 83 targeted sites demonstrating an ablation response (24 AF termination and 49 CL slowing). Sixty‐one out of 73 (83.6%) ESA were also identified on the sequential STAR maps. These showed greater consistency (*P* < .001), were seen pre‐ and post‐PVI (*P* < .001) and were more likely to be associated with AF termination on ablation (*P* = .007). The sensitivity and specificity of sequential mapping for the detection of ESA with an ablation response was 84.9% (95% confidence interval [CI] = 74.6‐92.2) and 90.0% (95% CI = 55.5‐99.8), respectively. During a follow‐up of 19.4 ± 3.7 months, 28 (80%) patients were free from AF/atrial tachycardia.

**Conclusions:**

STAR mapping consistently identified ESA in all patients and the ablation response was compatible with ESA being driver sites. Mechanistically important ESA were successfully identified using sequential recordings.

## 1 INTRODUCTION

Global atrial mapping to identify localized drivers in persistent atrial fibrillation (AF) has formed the foundation for several mapping systems,^1^, ^2^, ^3^, ^4^ which utilize either basket catheters or noninvasive surface electrocardiogram recordings. When dealing with the constantly changing cycle length (CL) of AF there is a clear advantage in achieving simultaneous atrial mapping. However, this does have its limitations including limitations to atrial coverage and contact achieved with basket catheters and the need for additional mapping tools that can result in additional costs and may require additional operator experience. The potential to map localized drivers in AF sequentially has been explored epicardially^5^ and endocardially.^6^

The Stochastic Trajectory Analysis of Ranked signals (STAR) system is a novel‐mapping method that compares activation times of unipolar electrograms in a dynamic fashion across mapping electrodes.^7^ There are two components incorporated in the method: (a) stochastic trajectory analysis, whereby the predominant direction of wavefront activation is determined and (b) ranking of signals, to determine how often the electrograms at each electrode leads relative to its neighbors. Electrodes that lead relative to their neighbors are then identified as early sites of activation (ESA). The STAR mapping method has been validated in vivo and in human studies mapping atrial tachycardias (ATs).^7^ It has also been used to guide ablation in persistent AF using global mapping with basket catheters, which has resulted in a significant response to ablation with targeting of ESA and high freedom from AF/AT during follow‐up.^8^

We hypothesized that the STAR mapping method could be applied in persistent AF using sequential mapping through demonstrating that ESA detected and ablated using global STAR maps could also effectively be identified using sequential mapping. The STAR sequential maps were also validated in mapping ATs where the mechanism was confirmed with conventional mapping, entrainment, and response to ablation.

## 2 METHODS

Patients undergoing catheter ablation for persistent AF (<24 months and no previous AF ablation) and AT were prospectively included. Patients provided informed consent for their study involvement. The study was approved by the UK National Research Ethics Service (16/LO/1379) and prospectively registered on http://clinicaltrials.gov (NCT02950844).

### 2.1 STAR mapping method

The STAR mapping method has been described in detail previously^7^, ^8^ (Supporting Information Method). In brief, the principle of the STAR mapping method is to use data from multiple individual wavefront trajectories to identify atrial regions that most often precede activation of neighboring areas, termed ESA. For a leading site to be classified as an ESA it was required to be leading for at least 75% of the time.

Global and sequential STAR maps were created using basket (Constellation; Boston Scientific, Natick, MA or FIRMap Abbott, CA) and PentaRay catheter unipolar recordings respectively. In this setting, the activation times are compared between electrode pairs that are within a predefined geodesic distance 8 ensuring that the electrodes are mapping the same anatomical surface and avoids ascribing a relationship between two regions that are separated by a distance and activated by unrelated wavefronts. For both AT and AF cases, the basket catheter was then removed and conventional multipolar catheters were used to create sequential STAR maps. For sequential mapping, activation times were compared across all the electrodes of a 20‐pole 2‐6‐2 mm spacing PentaRay catheter (Biosense Webster, Inc, CA) at each position. Care was taken to ensure the best possible electrode contact and spacing between the PentaRay catheter splines. Importantly, the activation times were compared within each recording taken rather than across all the recordings due to the sequential nature of the recordings. Each sequential PentaRay recording was then superimposed on the same geometry to produce an “amalgamation map” of separate recordings.

### 2.2 Electrophysiology procedure

All cases were performed with CARTO (Biosense Webster, Inc). Left atrial (LA) geometries and detailed bipolar voltage maps were created using the PentaRay catheter with a color fill threshold of 5 mm aiming for complete LA coverage.

#### 2.2.1 AT cohort

All patients were mapped in AT with a basket catheter as described for this cohort previously.^7^ The basket was then removed and at least 10 PentaRay recordings was performed. Following this, conventional Local activation time (LAT) maps were created and entrainment performed to elicit the AT mechanism. The mechanism was then confirmed by the response to ablation.

#### 2.2.2 AF cohort

A minimum of two 5‐minute basket catheter recordings were acquired to create global STAR maps (Supporting Information Method) pre‐ and post‐pulmonary vein isolation (PVI) as described for this cohort previously.^8^ A minimum of 10 recordings each of 30 seconds was acquired with the PentaRay catheter to achieve adequate LA coverage. The study aim was to map the LA but right atrial (RA) mapping was allowed if the coronary sinus activation was predominantly proximal to distal or the CL at the LA septum was persistently faster than the LA appendage.

The unipolar electrograms were recorded through Bard (Labsystem Pro Electrophysiology System). A decapolar catheter (Biosense Webster) positioned in the IVC was used as the indifferent catheter and electrograms were filtered between 0.5 to 500 Hz. The unipolar recordings and anatomical location data for the basket and PentaRay catheter were imported into Matlab (Matlab 2017b; MathWorks, MA) and using a custom‐written script, the global and sequential STAR maps were created.

### 2.3 Validation of STAR sequential maps in AT

In the context of focal/micro‐reentrant ATs a single ESA would be elicited during simultaneous mapping with basket catheters. Macro‐reentrant circuits, in contrast, will not display single ESA as the percentage of time an electrode is leading relative to its peers will be dependent on the number of electrodes it is paired with ahead and behind of the wavefront. Therefore, in this setting it is required that the operators evaluate the direction of wavefront propagation using the arrows displayed on the STAR maps. A sequential STAR map during AT will consist of an amalgamation map made up of all individual sequential recordings. For each sequential recording there will be a leading‐edge, with electrodes at that edge consistently leading and arrows showing the direction of wavefront propagation. With multiple such recordings projected onto the geometry, the operator can elicit the AT mechanism.

The sequential STAR map created in AT were reviewed offline independently by two observers that were blinded to the procedural data to ensure that they could accurately elicit the AT mechanism from only the sequential STAR map.

### 2.4 Global and sequential STAR mapping in AF

All patients underwent wide area circumferential ablation (WACA) to achieve PVI using a Thermocool SmartTouch Surround Flow catheter (Biosense Webster, Inc). Following this, a 20‐minute waiting period was observed so that no delayed effect of ablation could affect rhythm or CL during ESA ablation. During this waiting period the basket and sequential mapping data were obtained and the data exported to create STAR maps. The post‐PVI global STAR maps were then used to guide further ablation. The ablation strategy has previously been described in detail. Ablation was performed at the identified ESA with further consolidating lesions surrounding this site intentionally avoiding line formation. All ESA on a STAR map were targeted in order of ranked priority whereby sites that were leading 100% of the time being targeted first followed by 90% etc. If multiple ESA with the same ranked priority were identified it was at the operator's discretion in what order to ablate the ESA.

Ablation was stopped if: (a) 5 minutes of ablation had been performed, (b) no signal remained at the ablation site, or (c) a study‐defined ablation response had been achieved. Ablation responses were defined as the termination of AF (into AT or sinus rhythm), or CL slowing of ≥30 ms. Our group has previously applied this definition.^2^, ^3^ AF CL was measured over 30 beats from the PentaRay catheter positioned in the LA appendage. AF CL pre‐ and post‐ESA ablation was used to assess the ablation response. If AF terminated before some ESA had been ablated, these sites were not empirically targeted. No ablation beyond targeting ESA was allowed in AF. If AF organized into an AT this was mapped and ablated. Direct current (DC) cardioversion was performed at the end of the procedure if AF did not terminate following ablation of all identified ESA.

ESA that were associated with a study‐defined ablation response (AF termination or CL slowing ≥30 ms) were correlated offline to ESA identified on the sequential STAR maps. The ESA identified using each mapping modality were superimposed on the same geometry to ensure they had the same anatomical location. A custom written Matlab script was also used to elicit the distance between each site. The sites were required to be within 5 mm of each other to count as colocating. Two observers who were blinded to the procedural data reviewed and interpreted the sequential STAR maps to confirm the anatomical location of the ESA. The interoperator variability was recorded and differences were then resolved by consensus with a third blinded observer.

The characteristics of ESA that were seen on the global and sequential STAR maps were determined which included: (a) Ablation response achieved, (b) Consistency—defined as the proportion of global maps that showed the same ESA and (c) Identification of the ESA on pre‐ and post‐PVI maps.

Post hoc offline analysis of shorter time windows was also performed whereby the 5 minutes of unipolar electrogram recording collected post‐PVI using the basket catheter was split into 10 30‐seconds unipolar recordings. Each of these 30‐second unipolar recording was used to create separate consecutive STAR maps. These STAR maps were reviewed to determine if ESA that were prospectively targeted with ablation were consistently identified across all of these maps. The proportion of the ESA that were identified across the separate 30‐second recordings and that were identified on the sequential maps was determined to ensure the recording duration did not impact the identification of ESA across the two mapping modalities.

ESA identified on longer recordings and targeted prospectively with ablation were also assessed on each individual 30‐second segment to assess their consistency and to determine boundaries for recording time.

### 2.5 Follow‐up

All patients underwent clinical follow‐up at 3, 6, and 12 months, with 48‐hour ambulatory Holter monitoring at 6 and 12 months. On the clinician's discretion and patient's preference, patients could be followed‐up beyond the 12‐month period. A 3‐month “blanking period” was observed, with all medication including antiarrhythmic drugs continued during this time. Clinical success was defined as freedom from AF/AT lasting more than 30 seconds off antiarrhythmic drugs as per consensus recommendations.^9^

### 2.6 Statistical analysis

Statistical analyses were performed using SPSS (IBM SPSS Statistics, version 24 IBM Corp, NY) (Supporting Information Method). Continuous variables are displayed as mean ± standard deviation or median (range). Categorical variables are presented as a number and percentage. The Student *t* test or Mann‐Whitney *U* test was used for comparison of continuous variables. Sensitivities and specificity were calculated to determine the accuracy of sequential STAR maps in identifying ESA with a study‐defined ablation response. *P* < .05 was deemed as significant.

## 3 RESULTS

**Figure 1:**
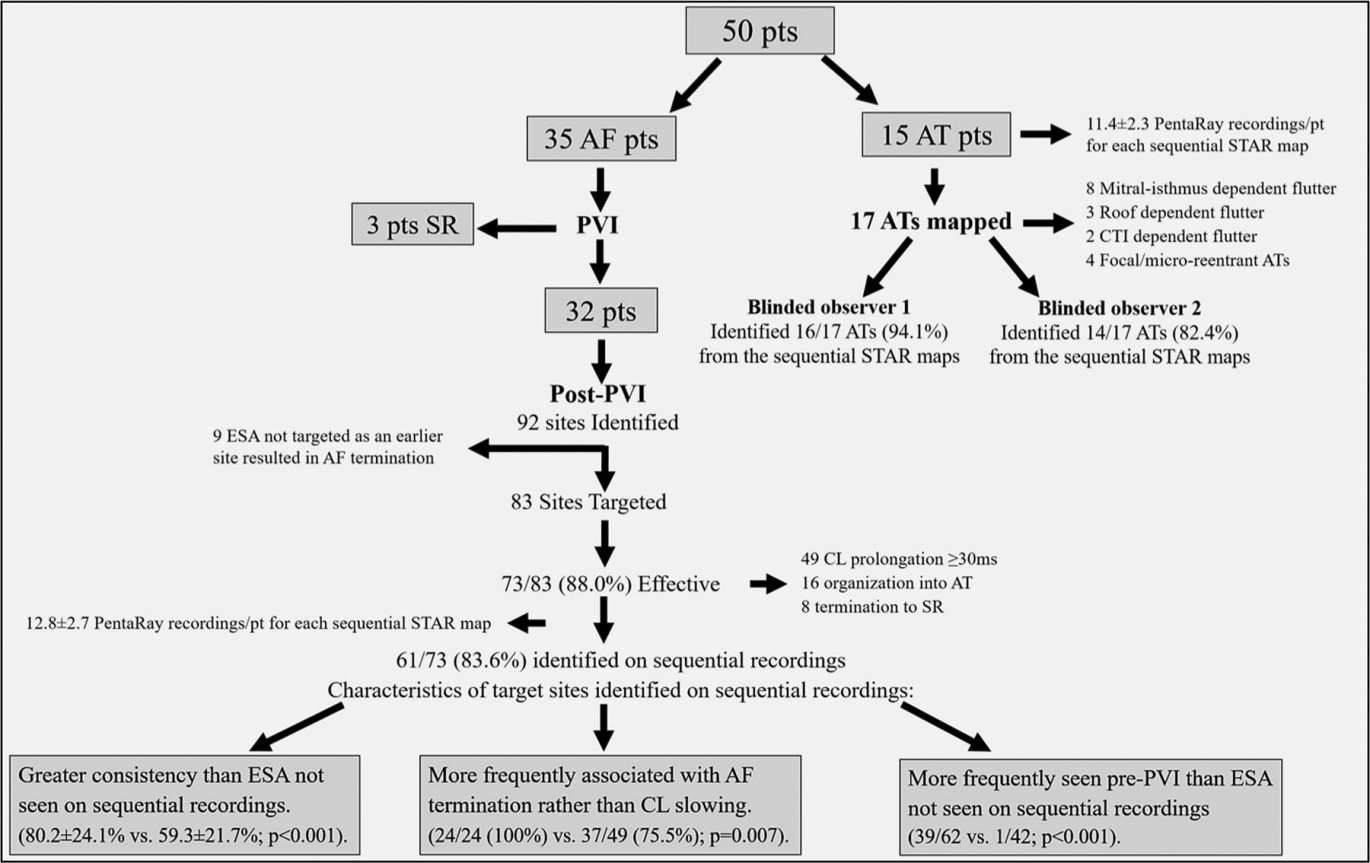
CONSORT flow diagram summarizing procedural outcomes

Fifty patients were included. The outcome of STAR mapping through panoramic recordings with basket catheters has been reported previously for the 15 patients undergoing mapping of AT,^7^ and for the 35 patients undergoing mapping of AF^8^ (Figure 1). Baseline characteristics are demonstrated in Table 1. One patient experienced cardiac tamponade which was noted at the end of the procedure which required pericardiocentesis. No other complications were encountered.

**Table 1:** Patient demographics

Baseline characteristics	AT patients n = 15	AF patients n = 35
Age (y), mean ± SD	61.9 ± 14.5	60.9 ± 9.4
Male n (%)	9 (60.0)	24 (68.6)
Diabetes mellitus n (%)	0	0
Hypertension n (%)	8 (53.3)	11 (31.4)
TIA/CVA n (%)	0	1 (2.9)
Ischemic heart disease n (%)	1 (6.7)	2 (5.7)
Cardiac surgery n (%)	1 (6.7)	1 (2.9)
Left ventricular EF ≥ 55% n (%)	14 (93.3)	31 (88.6)
LA area (cm^2^), mean ± SD	24.4 ± 4.1	26.5 ± 4.1
Bipolar voltage (mV), mean ± SD	0.29 ± 0.10	0.41 ± 0.17
AF duration (mo), mean ± SD	…	14.4 ± 5.3
Previous ablation n (%)		
AF	14 (93.3)	0
Cavotricuspid isthmus‐dependent flutter	2 (13.3)	2 (5.7)
Current medical strategy		
Calcium channel blocker	1 (6.7)	2 (5.7)
Beta‐blockers including Sotalol	14 (93.3)	17 (48.6)
Amiodarone	0	21 (60.0)
Flecainide	1 (6.7)	1 (2.9)
Current anticoagulation strategy		
Warfarin	11 (73.3)	4 (11.4)
Direct‐acting oral anticoagulants	4 (26.7)	31 (88.6)

Abbreviations: AF, atrial fibrillation; AT, atrial tachycardia; CVA, cerebrovascular attack; EF, ejection fraction; TIA, transient ischemic attack.

### 3.1 Validation of sequential STAR maps

Seventeen ATs were mapped in the 15 AT patients (Table 2). This included 13 macro‐reentrant AT and 4 focal/micro‐reentrant AT. An average of 11.4 ± 2.3 sequential recordings were taken per patient which ensured adequate LA coverage. Out of 17 ATs mapped, blinded observer 1 was able to accurately elicit the diagnosis of 16 ATs from the sequential STAR maps (94.1%). The one AT that was not identified by the blinded observer 1 was a mitral isthmus‐dependent flutter however, this observer was able to elicit the diagnosis of 7 mitral isthmus‐dependent flutters in other patients from the sequential STAR maps. Blinded observer 2 was able to accurately elicit the diagnosis of 14 ATs from the sequential STAR maps (82.4%). Blinded observer 2 was unable to elicit the diagnosis of 2 mitral isthmus‐dependent flutters and 1 roof‐dependent flutter. However, blinded observer 2 was able to elicit the diagnosis of 6 and 2 mitral isthmus‐ and roof‐dependent flutters respectively in other patients.

**Table 2:** Mechanism of the ATs mapped

ATs mapped and ablated	
AT n %	17
Macro‐reentrant	13 (76.5)
Mitral isthmus‐dependent flutter	8 (47.1)
Roof‐dependent flutter	3 (17.6)
Cavotricuspid isthmus‐dependent flutter	2 (11.8)
Focal/micro‐reentrant	4 (23.5)
LA mid anterior	1 (5.9)
LA mid roof	1 (5.9)
Right focal/micro‐reentrant	1 (5.9)
Ligament of Marshall	1 (5.9)

Abbreviations: AT, atrial tachycardia; LA, left atrial.

### 3.2 Global and sequential STAR mapping in AF

Thirty‐five patients were included (Figure 1 and Table 1). The mean age was 60.9 ± 9.4 years with 24 patients (68.6%) being male. The mean AF duration was 14.4 ± 5.3 months (n = 7 AF duration < 12 months and n = 28 AF duration > 12 months).

#### 3.2.1 ESA with an ablation response on global STAR maps

Of the 35 patients, three patients terminated to sinus rhythm with PVI and therefore post‐PVI ESA were not identified or targeted in these patients. Offline analysis in the three patients in whom AF termination was achieved during PVI, ESA were identified at the PV ostium on the site of the WACA lines, and these ESA we colocated at the sites where AF terminated during PVI.

In the remaining 32 patients, 92 ESA were identified post‐PVI of which 83 (90.2%, 2.6 ± 0.8 per patient) ESA were targeted with ablation. The nine ESA that were not ablated were in patients in whom ablation at a previous site had resulted in AF termination. A study‐defined ablation response was seen in all patients which included 73 (88.0%) of the ESA, with an average of 2.3 ± 0.6 ESA sites per patient.

**Figure 2:**
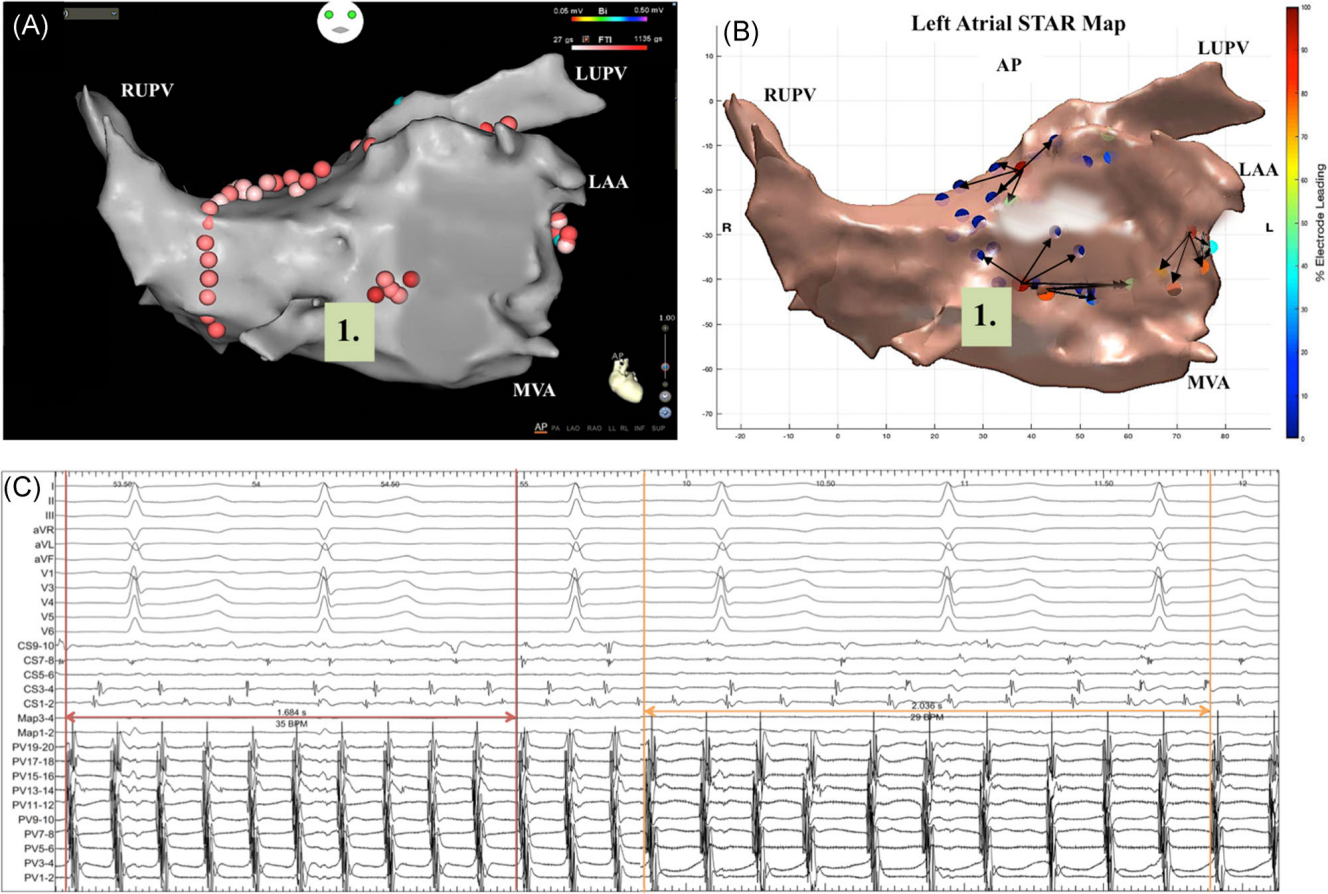
A‐C, Patient ID 9. Demonstrates (A) CARTO LA map in an anterior‐posterior view that shows localized ablation for 2.2 minutes at the low anterior wall post‐PVI which was guided by (B) a global STAR LA map in an anterior‐posterior view that shows an ESA mapped to this area (highlighted by the number 1). C, As shown on the electrograms obtained from BARD ablation here resulted in CL slowing from 168 to 203 ms when measured across 10 beats with the PentaRay catheter positioned in the LA appendage. In the study, 30 beats were used to assess CL slowing however, for figure illustration, 10 beats were used to allow greater electrogram resolution. The global STAR map also highlights two further ESA, one mapped to the high lateral wall and one mapped to the roof. Ablation at the ESA on the lateral wall resulted in AF termination to sinus rhythm. The ESA on the roof was therefore not targeted. AF, atrial fibrillation; CL, cycle length; ESA, early sites of activation; LA, left atrial; LAA, left atrial appendage; LUPV, left upper pulmonary vein; MVA, mitral valve annulus; PVI, pulmonary vein isolation; RUPV, right upper pulmonary vein; STAR, Stochastic Trajectory Analysis of Ranked

**Figure 3:**
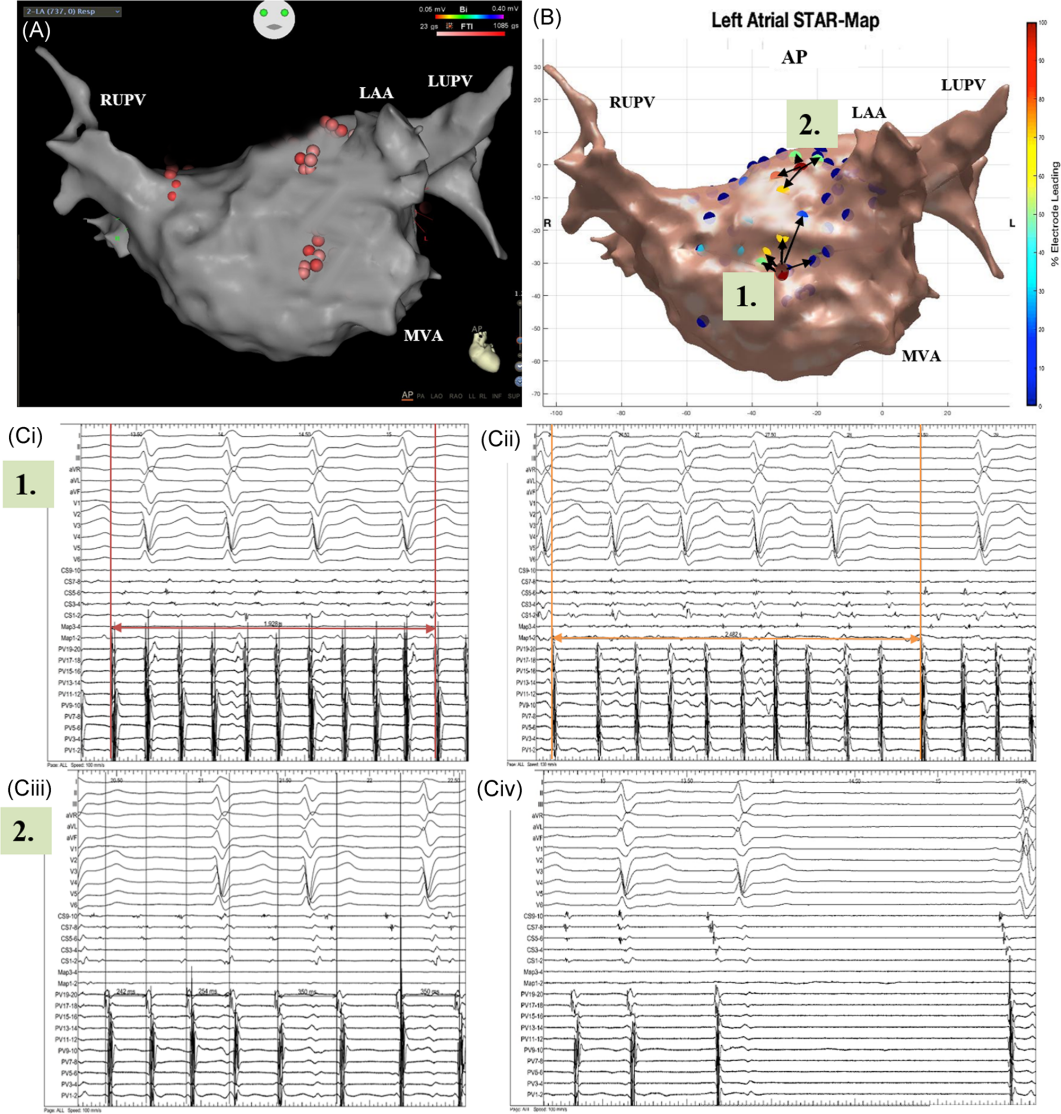
A‐C, Patient ID 11. Demonstrates (A) a CARTO LA map in an anterior‐posterior view that shows WACAs to achieve PVI and cluster ablation at mid anterior wall and roof as guided by (B) a global STAR LA map in an anterior‐posterior view which highlights two ESA, (1) mid anterior wall (highlighted by number 1) (2) roof (highlighted by number 2) (Ci‐iv) where ablation for (i‐ii) 3.0 minutes resulted in CL slowing (from 198 to 248 ms) with ablation of the first ESA and (iii‐iv) 2.2 minutes at second ESA resulted in AF termination to AT which was ablated and sinus rhythm achieved as shown on the BARD electrograms. AF, atrial fibrillation; AT, atrial tachycardia; CL, cycle length; ESA, early sites of activation; LA, left atrial; LAA, left atrial appendage; LUPV, left upper pulmonary vein; MVA, mitral valve annulus; PVI, pulmonary vein isolation; RUPV, right upper pulmonary vein; STAR, Stochastic Trajectory Analysis of Ranked; WACA, wide area circumferential ablation

On a per ESA basis, AF termination was achieved with ablation at 24 sites (16 organization to AT and 8 termination to sinus rhythm) and CL slowing of ≥30 ms was achieved with 49 sites (Figure 2A‐C, Figure 3A‐D and Table S1). On a per patient basis 24 patients had AF termination (75%) and 8 patients had CL slowing (25%) on ablation of ESA. All ATs were effectively ablated to achieve sinus rhythm at the end of the procedure. Eight patients who remained in AF underwent DC cardioversion at the end of the procedure.

#### 3.2.2 ESA with an ablation response identified on sequential STAR maps

The 32 patients had on average 12.8 ± 2.7 sequential recordings performed. The two observers analyzing sequential STAR maps offline (blinded to other procedural information and to the global STAR maps) identified 65 of the 92 ESA (70.7%) identified on the global STAR maps. Kappa was 0.81 (95% confidence interval [CI] = 0.60‐1.00) indicating a strong interobserver agreement.

**Figure 4:**
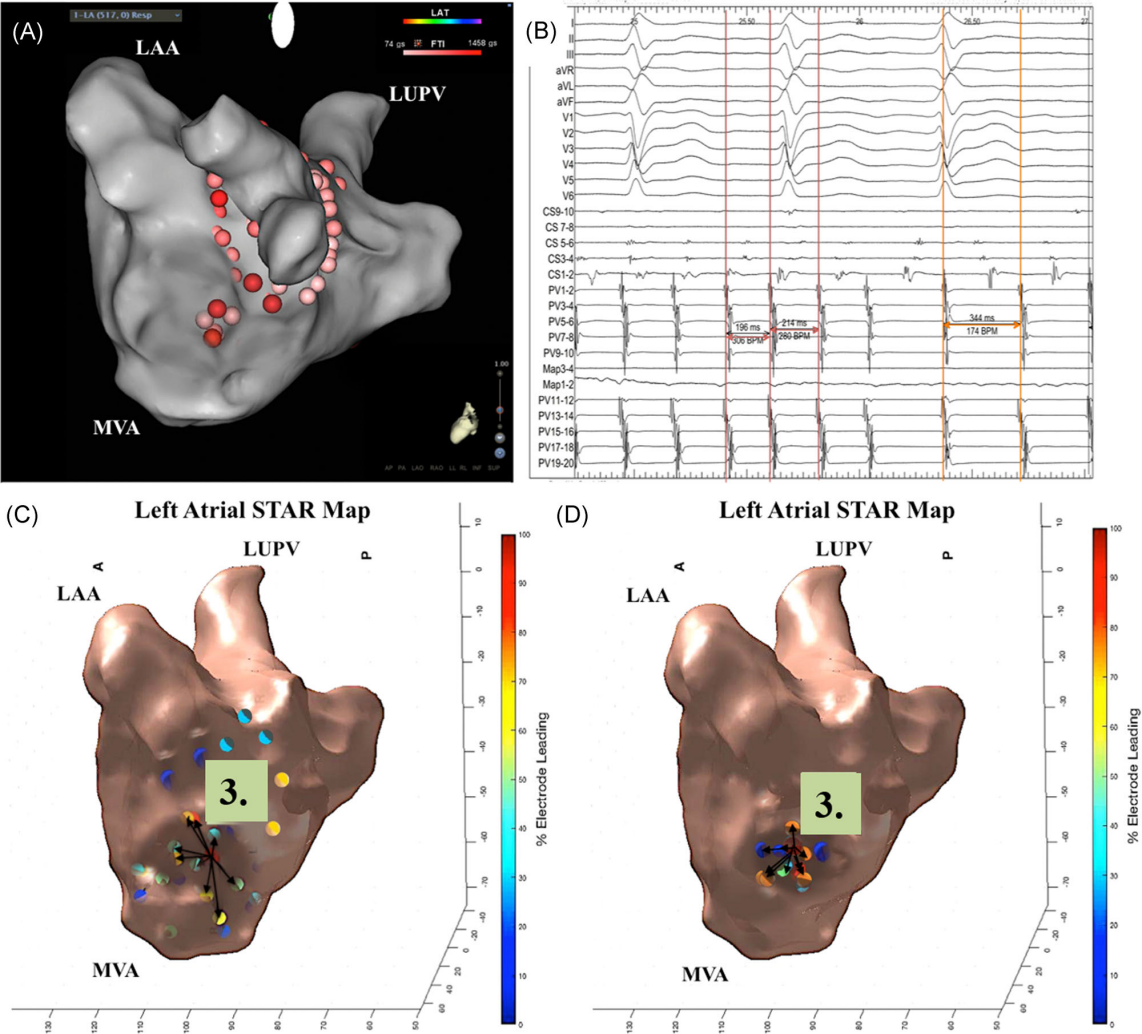
A‐D, Patient ID 32. Demonstrates (A) a CARTO LA map in a tilted lateral view where 3.0 minutes of ablation resulted in (B) AT as shown on the electrograms obtained from BARD. (C) The ablation was guided by the global STAR LA map in a lateral view that shows an ESA. The electrograms obtained at the ESA as highlighted by an asterix is leading neighboring electrodes. The red cross highlights the site of annotation of the atrial electrograms by the STAR mapping method. (D) A sequential STAR LA map in a lateral view demonstrates the same ESA and again the electrograms obtained at the ESA is leading neighboring electrodes. AT, atrial tachycardia; ESA, early sites of activation; LA, left atrial; LAA, left atrial appendage; LUPV, left upper pulmonary vein; MVA, mitral valve annulus; STAR, Stochastic Trajectory Analysis of Ranked

**Figure 5:**
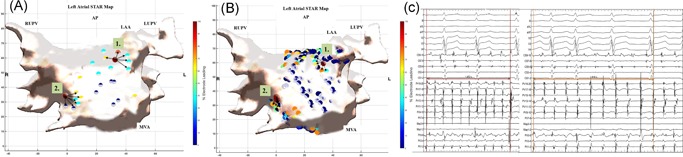
A‐C, Patient ID 22. Demonstrates (A) a global STAR map in anterior‐posterior view highlighting two ESA. Ablation of the ESA mapped to the anterior LAA (highlighted by number 1) resulted in CL slowing. The ESA mapped to the anteroseptum (highlighted by number 2) also resulted in CL slowing on ablation. B, Amalgamation STAR map of eight sequential PentaRay recordings in anterior‐posterior view highlighting the same two ESA that were identified on the global STAR map. C, BARD signals demonstrating CL slowing from 145 to 182 ms on ablation at the ESA at the anteroseptum. CL, cycle length; ESA, early sites of activation; LAA, left atrial appendage; LUPV, left upper pulmonary vein; MVA, mitral valve annulus; RUPV, right upper pulmonary vein; STAR, Stochastic Trajectory Analysis of Ranked

Out of the 83 ESA that were identified and ablated based on the global STAR maps, 62 were identified on the sequential STAR maps (74.7%). However, when only assessing the 73 ESA that were associated with an ablation response 61 (83.6%) were identified on the sequential STAR maps (Figure 4A‐D and Figure 5A‐C). The sites were on average 2.1 ± 1.2 mm from each other on the coregistered maps. Only 1 out of the 10 ESA that were not associated with an ablation response were seen on the sequential STAR maps.

ESA with an ablation response were more frequently identified on the sequential maps if the ablation response was AF termination rather than CL slowing (24/24 [100%] vs 37/49 [75.5%]; *P* = .007). ESA that were identified on global and sequential maps showed greater consistency compared to the ESA that were only seen on the global maps (ESA observed on 80.2% ± 24.1% vs 59.3% ± 21.7% of global STAR maps created in each patient; *P* < .001). These ESA were also more frequently seen on both pre‐ and post‐PVI compared to ESA not seen on sequential maps (38/61 [62.3%] ESA seen on sequential maps were seen both pre‐ and post‐PVI vs 1/12 [8.3%] ESA not seen on sequential maps were seen on pre‐and post‐PVI maps; *P* < .001).

On the sequential STAR maps, 12 ESA were identified that were not seen on the global STAR maps. When reviewing the global STAR maps, the anatomical surface to which these ESA were mapped correlated to sites with poor basket catheter coverage and contact in 10 of these 12 ESA and 8 of these occurred in patients where CL slowing was achieved. The remaining two ESA identified on the sequential STAR maps occurred at sites with good basket coverage and contact yet still without evidence of ESA on the global STAR maps. Therefore, if the 10 ESA identified on sequential mapping at sites essentially unmapped with the basket due to poor coverage were disregarded, the sensitivity and specificity of sequential mapping for the detection of ESA with an ablation response was 84.9% (95% CI = 74.6‐92.2) and 90.0% (95% CI = 55.5‐99.8) respectively.

The 5‐minutes STAR map recordings consisted in total of 860 30‐second segments. Of the 83 ESA that were identified and ablated, 74 (89.1%) were consistently identified across the 30‐second segments of which 60 (81.1%) were identified on the sequential maps.

### 3.3 Follow‐up data

During an average follow‐up of 19.4 ± 3.7 months (all patients reached ≥12 months follow‐up), 28 patients (80%) were free from AF/AT, off antiarrhythmic drugs. Of the seven patients that had recurrent AF/AT during follow‐up, six had documented AT of which all underwent successful AT ablation.^8^

## 4 DISCUSSION

This study describes the application of a novel statistical vector mapping methodology using consecutive electrogram recordings with multipolar catheters. STAR mapping of complex wavefront movement was successfully validated in AT, with the mechanism successfully identified in a majority of cases. We previously described the identification of ESA in all patients in this cohort (usually 2‐3) using basket catheters and the ablation response and freedom from AF/AT suggests they are plausible drivers in persistent AF.^8^ A majority of ESA identified on global mapping were also identified through sequential mapping, showing consistency in their identification between mapping modalities. ESA that were identified on consecutive mapping were more consistent (ie, were identified on a higher proportion of global basket maps) than ESA not detected on consecutive mapping. All ESA that resulted in AF termination were successfully detected with consecutive mapping. In fact, sequential mapping identified ESA that were not detected with basket mapping due to areas with poor coverage. A vector mapping approach to AF is therefore feasible with either basket and sequential mapping and each may have advantages in identifying drivers.

### 4.1 Use of sequential STAR mapping to elicit AT mechanisms

The STAR mapping method using global STAR maps has previously been validated in paced beats and in AT.^7^ In this study, it has been shown that sequential STAR maps can be used to elicit the diagnosis of macro‐reentrant and focal/micro‐reentrant ATs in the RA and LA. By examining an amalgamation map of sequential PentaRay recordings, operators were able to interpret complex wavefront movements to successfully determine the mechanism of a majority of these AT. STAR mapping was applied to patients with AT with the aim of validating this mapping approach in terms of mapping complex wavefronts where the mechanism can be confirmed. However, STAR mapping could have a potentially useful clinical role in this context.

Conventional LAT mapping is dependent on having a fixed reference point. In the context of an AT with a variable CL or changing mechanism, an operator can be faced with the need to re‐map repeatedly and may have difficulty in eliciting the AT mechanism. The STAR mapping method is based on comparing activation times across several electrodes in a dynamic fashion over the whole recording to identify sites that are predominantly leading in relation to other poles. As a result, it is not dependent on a fixed reference or even an entirely consistent mechanism. This vector mapping approach may, therefore, lend itself well to ATs associated with a changing CL or potentially a changing mechanism.

### 4.2 STAR mapping method

The STAR mapping method has been validated in vivo and in human studies mapping AT.^7^ Utilizing global STAR maps has also been successfully used to guide AF ablation which has been associated with high rates of AF termination and high rates of freedom from AF/AT during follow‐up.^8^

The definition used for localized drivers in AF continues to be debated with alternative definitions used in different studies.^1^, ^10^ There are data emerging from centers using different mapping technologies suggesting that AF may be sustained by focal and rotational sources that are spatially stable but with temporal periodicity.^1^, ^2^, ^3^, ^11^ However, it remains unclear how best to map and ablate these potential drivers. Data suggests that electrogram characteristics, fractionation, and frequency analysis do not reliably identify driver sites, suggesting that other methods of driver detection are needed.^3^, ^12^

The STAR mapping method utilizes a statistical vector mapping approach to identify sites of early activity in the atrium by comparing activation times. Unlike other systems, the STAR mapping method does not rely on the operator's interpretation of wavefront propagation maps to identify focal or rotational activation which may result in bias.

### 4.3 Use of sequential STAR maps to detect localized drivers in AF

This is the first study to report on a vector mapping approach using sequential mapping in AF. Sequential STAR maps were able to identify with high sensitivity and specificity the same ESA that were identified and ablated based on the global STAR maps. This amalgamation of several PentaRay recordings was interpreted consistently between different observers blinded to other data. ESA that were identified on sequential maps were shown to be more consistent and also more likely to be seen pre‐ and post‐PVI. Driver sites that are more consistent and are identified pre‐ and post‐PVI may be more likely to be associated with AF termination on ablation.^3^ This may be why all ESA associated with AF termination were identified on the sequential STAR maps. Therefore, the sequential approach to STAR mapping identified a majority of ESA that were identified with basket mapping, and perhaps, more importantly, it seemed to identify those that were most consistent and mechanistically important.

The additional ESA that were identified on the sequential STAR maps and not seen on the global maps were, in a majority of cases, at sites where poor basket catheter coverage and contact was achieved. Therefore, although these sites could represent false positives, given the consistency observed between ESA identified on basket and sequential mapping elsewhere it is perhaps more likely that these sites represent ESA that were missed by basket mapping. As a majority of these sites were encountered in patients where CL slowing was achieved rather than AF termination, it is unclear whether targeting these ESA would have terminated AF. Since sequential mapping seemed to identify most of the mechanistically important ESA, and had advantages in terms of coverage and mapping density compared to the basket, it is uncertain at present which modality is optimal for STAR analysis.

The STAR methodology is distinct from other AF mapping technologies reported to date. The ECGI system and Topera both utilize phase mapping. A recent report of electrographical flow mapping utilizes voltage measurements obtained from unipolar recordings to generate voltage shapes which can be visualized on maps to allow for the identification of rotors and focal drivers,^13^ which is distinct from STAR mapping which is based on electrode timings to generate a statistical vector map. Focal drivers have recently been demonstrated through sequential mapping using a “region of interest” algorithm within the CARTOFINDER mapping system.^6^ As the focus of that study was to map focal drivers it remains unclear whether sequential mapping would identify rotational drivers which may be less stable than focal drivers.^1^, ^2^, ^3^ As the STAR mapping method relies on identifying sites of early or leading activity rather than whether the driver site is focal or rotational, the differences in these mechanisms may not be important with this vector mapping approach. Indeed, there is arguably less interpretation needed of a color‐coded STAR map than an activation map or phase map seeking to identify a particular mechanism.

## 5 LIMITATIONS

One of the study limitations is the small patient numbers. At 50 patients, this is relatively large for a validation study and analysis of AF mechanisms, but small by the standards of a trial studying clinical outcomes. Having assessed 92 ESA, these data have allowed effective evaluation of the STAR mapping method in terms of identifying mechanistically important sites in AF and showed that these sites can be accurately identified with global and sequential STAR mapping. Further randomized studies are needed to determine the impact of this ablation strategy on clinical outcomes.

The study aim was to validate global and sequential STAR mapping in terms of its ability to identify ESA that were prospectively targeted on global STAR maps to determine the mechanistic significance of these sites. At the time these cases were conducted sequential STAR maps could not be applied prospectively in real‐time and hence ablation was guided by global basket catheter mapping. Further work is needed to target ablation prospectively using sequential STAR mapping to determine the effectiveness of this approach.

## 6 CONCLUSIONS

Consecutive mapping in AT demonstrated that the STAR mapping method can successfully map complex wavefront movements. In AF, a majority of ESA identified on global STAR mapping are also identified using sequential mapping. All important ESA (ie, those that resulted in AF termination) were successfully detected with consecutive mapping and in fact, sequential mapping identified ESA that were not detected with basket mapping due to areas with poor coverage. A vector mapping approach to AF is therefore feasible with either basket or sequential mapping and each may have advantages in identifying drivers. The ablation response at ESA and freedom from AF/AT suggests they are plausible drivers in persistent AF. Prospective randomized studies targeting these sites are needed to further evaluate the clinical impact.

## Supporting information

Supplementary information

Supplementary information

Supplementary information

Supplementary information
