# Supplementary material for: STAR mapping method to identify driving sites in persistent atrial fibrillation: Application through sequential mapping
Source: J Cardiovasc Electrophysiol. 2019 Oct 3;30(12):2694–703. doi: 10.1111/jce.14201 (PMC6916564; doi:10.1111/jce.14201)
Supplement: Supplementary file 1 — Supplementary information [file JCE-30-2694-s001.docx]

**Supplemental Figure Legend**

**Figure 1 A-C-** Local activation time (LAT) maps in **i)** anterior-posterior and **ii)** left lateral view that demonstrates a mitral isthmus dependent flutter of which mechanism was confirmed with entraintment and response to ablation. **Bi-ii)** STAR maps in an **i)** anterior-posterior view and **ii)** left lateral view that were created with basket catheter recordings demonstrating a mitral isthmus dependent flutter. **Ci-ii)** STAR maps in an **i)** anterior-posterior and **ii)** left lateral view that were created with PentaRay recordings demonstrating a mitral isthmus dependent flutter.
