# Supplementary material for: STAR mapping method to identify driving sites in persistent atrial fibrillation: Application through sequential mapping
Source: J Cardiovasc Electrophysiol. 2019 Oct 3;30(12):2694–703. doi: 10.1111/jce.14201 (PMC6916564; doi:10.1111/jce.14201)
Supplement: Supplementary file 2 — Supplementary information [file JCE-30-2694-s002.docx]

**SUPPLEMENTAL METHODS**

1. *STAR mapping method*

The principle of the STAR mapping method is to use data from multiple individual wavefront trajectories to identify regions of the atrium that most often precede activation of neighboring areas. By gathering data from many thousands of activations, a statistical model can be formed. This permits regions of the atrium to be ranked according to the amount of time that activations precede those of adjacent regions. Unipolar activation timing was taken as the maximum negative deflection (peak negative dv/dt). Through utilizing pre-defined refractory periods ^9^ the mapping method avoids assigning activations from separate wavefronts or fractionated electrograms. Electrode timing relationships that are implausible due to conduction velocity restraints are also excluded by the mapping method (7).

A STAR map consists of color-coded electrode positions projected on to patient’s atrial geometry which was exported from CARTO. Each color represents the proportion of time the electrode spent leading in relation to the other paired electrodes as highlighted by the color scale on the right hand side of the STAR maps.

1. *Creation of STAR maps*

The unipolar electrograms were recorded through Bard (Labsystem pro Electrophysiology system). A decapolar catheter (Biosense Webster, CA) positioned in the IVC was used as the indifferent catheter and electrograms were filtered between 0.5-500Hz. The unipolar recordings and anatomical location data for the basket and PentaRay catheter were exported from CARTO and imported into Matlab (Matlab 2017b, MathWorks MA, USA). Using a custom written script the global and sequential STAR maps were created.
