# Supplementary material for: STAR mapping method to identify driving sites in persistent atrial fibrillation: Application through sequential mapping
Source: J Cardiovasc Electrophysiol. 2019 Oct 3;30(12):2694–703. doi: 10.1111/jce.14201 (PMC6916564; doi:10.1111/jce.14201)
Supplement: Supplementary file 3 — Supplementary information [file JCE-30-2694-s003.docx]

*Supplemental Table 1- Demonstrates the number of ESA identified and ablated in each patient and the achieved ablation response on a per ESA basis.*

| **Patient ID** | **Number of ESA^*^ identified** | **Number of ESA ablated** | **Anatomical location of ESA** | **Visible on pre-PVI^Ͳ^ maps** | **Proportion of post-PVI maps that identify the ESA ablated %** | **Identified on sequential STAR maps** | | **Ablation response** | | **Ablation duration min** |
| --- | --- | --- | --- | --- | --- | --- | --- | --- | --- | --- |
| 1 | 3 | 3 | Low anterior  High anterior  Endocardial proximal CS | Yes  Yes  No | 50  50  50 | | Yes SCL  Yes SCL  Yes SCL | | 2.3  2.8  2.1 | |
| 2 | 2 | 2 | Roof/LAA  Mid anterior | No  No | 66  66 | | No No effect  No SCL | | 1.8  2.5 | |
| 3 | 4 | 4 | Mid roof  Mid posterior  Low anterior  Septal RA | Yes  Yes  No  No | 100  100  100  100 | | No No effect  Yes SCL  No SCL  No SCL | | 2.1  3.1  3.0  2.5 | |
| 4 | 4 | 4 | Mid Roof  Mid Posterior  Low anterior  Lateral RA | No  Yes  Yes  Yes | 100  100  100  100 | | No No effect  Yes SCL  Yes SCL  Yes SCL | | 2.2  3.0  2.4  2.5 | |
| 5 | 2 | 2 | Lateral inferior  High anterior | No  Yes | 50  100 | | Yes SCL  Yes AT | | 2.8  3.1 | |
| 6 | 2 | 2 | Low anterior  Mid roof | No  Yes | 33  100 | | No SCL  Yes SR | | 3.0  1.9 | |
| 7 | 2 | 2 | Posterior septal  Anterior LAA | No  Yes | 66  66 | | No No effect  Yes SCL | | 2.1  3.5 | |
| 8 | 2 | 2 | Anterior LAA  Septal RA | Yes  Yes | 66  66 | | Yes SCL  Yes SCL | | 3.1  2.0 | |
| 9 | 3 | 2 | Low anterior  High lateral  Roof | Yes  Yes  No | 75  75  50 | | Yes SCL  Yes SR  No Not targeted | | 2.2  1.8 | |
| 10 | 2 | 2 | Roof/LAA  Lateral | No  No | 100  66 | | Yes SCL  No SCL | | 2.5  2.4 | |
| 11 | 2 | 2 | Mid anterior  Roof | No  Yes | 50  100 | | No SCL  Yes AT | | 3.0  2.2 | |
| 12 | 4 | 3 | Lateral  Inferior RLPV  Mid posterior  Mid anterior | No  No  Yes  No | 100  50  100  50 | | No SCL  No No effect  Yes SR  No Not targeted | | 2.0  1.9  2.5 | |
| 13 | 2 | 2 | LAA/Roof  Inferior | No  Yes | 33  100 | | Yes SCL  Yes AT | | 2.1  1.9 | |
| 14 | 3 | 2 | High anterior  Mid Posterior  Roof | Yes  Yes  Yes | 100  100  66 | | Yes SCL  Yes SR  Yes Not targeted | | 2.3  2.0 | |
| 15 | 4 | 3 | Roof/LAA  Inferior to RUPV  Postero-inferior  Antero-septum | Yes  No  Yes  Yes | 100  33  100  33 | | Yes SCL  No No effect  Yes AT  Yes Not targeted | | 2.1  1.9  1.8 | |
| 16 | 3 | 3 | High anterior  Postero-lateral  Lateral | No  No  Yes | 100  100  100 | | Yes SCL  Yes SCL  Yes AT | | 2.5  2.4  3.9 | |
| 17 | 3 | 3 | LAA  Distal endocardial CS  LAA/Roof | No  No  Yes | 100  100  100 | | Yes SCL  Yes SCL  Yes AT | | 2.9  2.0  3.0 | |
| 18 | 2 | 2 | Lateral  Antero-septum | No  No | 50  50 | | Yes SCL  Yes SCL | | 2.5  2.7 | |
| 19 | 3 | 2 | Infero-lateral  Roof  Mid anterior | No  Yes  No | 66  100  66 | | No SCL  Yes AT  No Not targeted | | 2.2  3.1 | |
| 20 | 2 | 2 | Lateral  Roof | No  Yes | 50  100 | | Yes SCL    Yes AT | | 2.3  3.1 | |
| 21 | 2 | 1 | Antero-septum  Mid posterior | Yes  No | 100  33 | | Yes SR  No Not targeted | | 2.8 | |
| 22 | 3 | 3 | Anterior LAA  Antero-septum  Postero-lateral | No  No  Yes | 100  100  100 | | Yes SCL  Yes SCL  Yes AT | | 2.1  3.2  2.5 | |
| 23 | 3 | 3 | Mid Roof  Antero-septum  Mid posterior | Yes  No  Yes | 100  100  100 | | Yes SCL  Yes SCL  Yes AT | | 2.8  1.9  3.4 | |
| 24 | 3 | 3 | Mid posterior  Endocardial distal CS  Mid roof | No  No  Yes | 33  33  100 | | No No effect  Yes SCL  Yes AT | | 4.5  2.2  2.7 | |
| 25 | 4 | 4 | Mid anterior  Roof  Mid posterior  Antero-septum | Yes  No  No  Yes | 33  33  33  100 | | Yes SCL  Yes No effect  Yes SCL  Yes AT | | 2.5  3.0  2.1  2.5 | |
| 26 | 4 | 4 | Endocardial proximal CS  High posterior  Anterior roof  High anterior | No  No  No  No | 66  33  33  66 | | Yes SCL  Yes SCL  No No effect  Yes AT | | 3.1  2.2  2.4  2.9 | |
| 27 | 2 | 2 | High posterior  Antero-septum  Base of LAA | Yes  Yes  No | 50  100  50 | | Yes SCL  Yes AT  No Not targeted | | 2.2  1.8 | |
| 28 | 3 | 3 | Lateral  Postero-septal  Mid anterior | No  No  Yes | 50  100  100 | | No No effect  Yes SCL  Yes AT | | 1.4  2.6  2.4 | |
| 29 | 3 | 3 | Inferior RUPV  Mid lateral  Mid posterior | No  No  Yes | 33  66  66 | | No SCL  Yes SCL  Yes AT | | 2.3  2.1  2.2 | |
| 30 | 3 | 2 | Roof  Mid anterior  Lateral | No  Yes  No | 66  100  66 | | No SCL  Yes SR  No Not targeted | | 2.5  3.2 | |
| 31 | 4 | 3 | LAA/Roof  Low anterior  Mid lateral  Mid anterior | Yes  No  Yes  No | 66  66  100  33 | | Yes SCL  No SCL  Yes AT  No Not targeted | | 2.8  2.4  3.0 | |
| 32 | 4 | 3 | Roof  Mid anterior  Mid lateral | No  No  Yes | 66  66  66 | | No SCL  Yes SCL  Yes AT | | 2.1  2.2  3.0 | |

^*^ESA- early site of activation

^Ͳ^PVI- pulmonary vein isolation
