# Supplementary figures and images for: STAR mapping method to identify driving sites in persistent atrial fibrillation: Application through sequential mapping
Source: J Cardiovasc Electrophysiol. 2019 Oct 3;30(12):2694–703. doi: 10.1111/jce.14201 (PMC6916564; doi:10.1111/jce.14201)

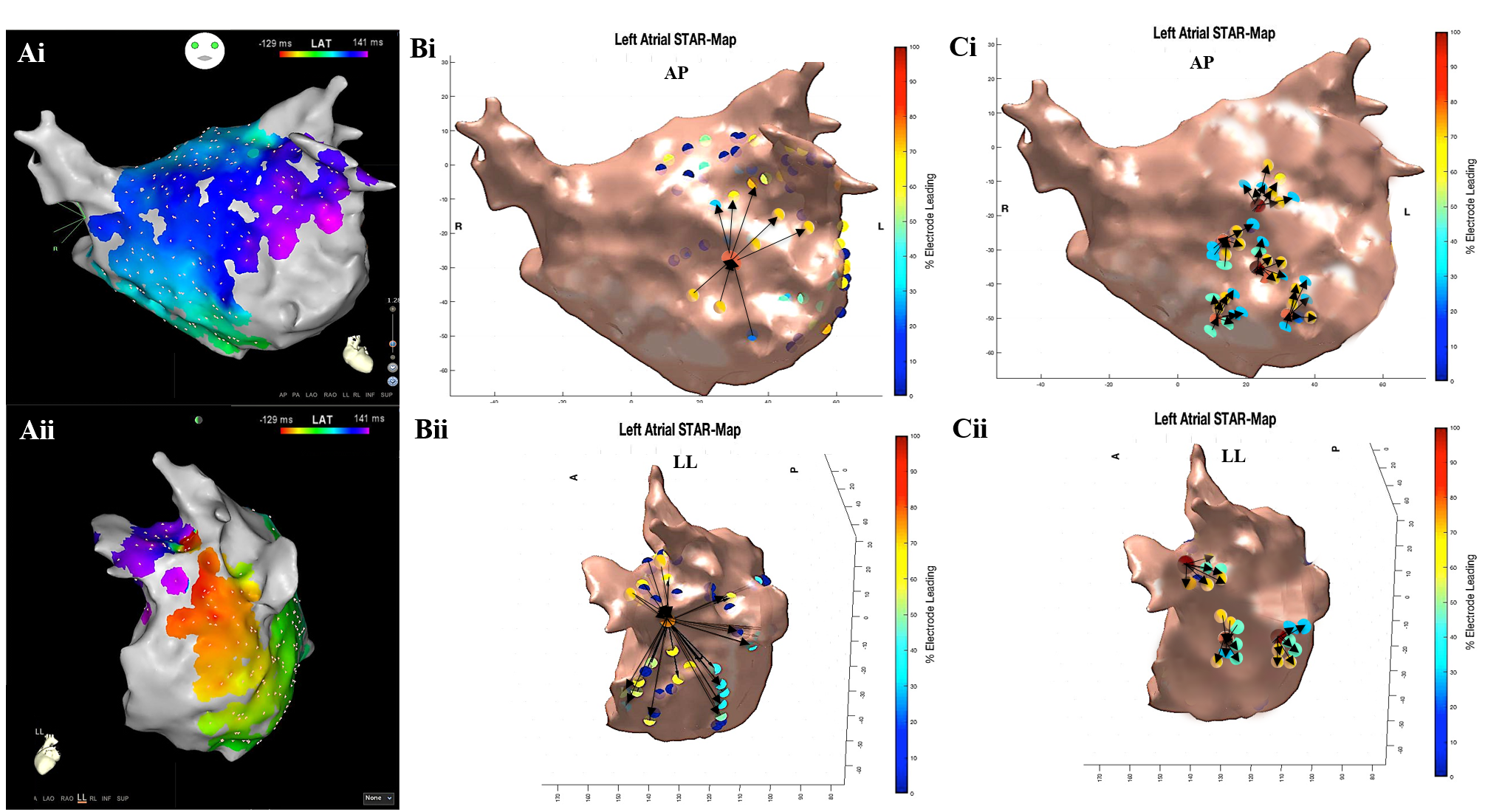

Supplement: Supplementary file 4 — Supplementary information [file JCE-30-2694-s004.tif]
